# Schizophrenia polygenic risk scores in youth mental health: preliminary associations with diagnosis, clinical stage and functioning

**DOI:** 10.1192/bjo.2021.14

**Published:** 2021-02-22

**Authors:** Jacob J. Crouse, Joanne S. Carpenter, Frank Iorfino, Tian Lin, Nicholas Ho, Enda M. Byrne, Anjali K. Henders, Leanne Wallace, Daniel F. Hermens, Elizabeth M. Scott, Naomi R. Wray, Ian B. Hickie

**Affiliations:** Youth Mental Health & Technology Team, Brain and Mind Centre, University of Sydney, Australia; Youth Mental Health & Technology Team, Brain and Mind Centre, University of Sydney, Australia; Youth Mental Health & Technology Team, Brain and Mind Centre, University of Sydney, Australia; Queensland Brain Institute, University of Queensland, Australia; and Institute of Molecular Bioscience, University of Queensland, Australia; Youth Mental Health & Technology Team, Brain and Mind Centre, University of Sydney, Australia; Institute of Molecular Bioscience, University of Queensland, Australia; Institute of Molecular Bioscience, University of Queensland, Australia; Institute of Molecular Bioscience, University of Queensland, Australia; Sunshine Coast Mind and Neuroscience Thompson Institute, University of the Sunshine Coast, Australia; St Vincent's and Mater Clinical School, The University of Notre Dame, Australia; Queensland Brain Institute, University of Queensland, Australia; and Institute of Molecular Bioscience, University of Queensland, Australia; Youth Mental Health & Technology Team, Brain and Mind Centre, University of Sydney, Australia

**Keywords:** Genetics, youth mental health, psychiatry, early intervention, transdiagnostic

## Abstract

**Background:**

The schizophrenia polygenic risk score (SCZ-PRS) is an emerging tool in psychiatry.

**Aims:**

We aimed to evaluate the utility of SCZ-PRS in a young, transdiagnostic, clinical cohort.

**Method:**

SCZ-PRSs were calculated for young people who presented to early-intervention youth mental health clinics, including 158 patients of European ancestry, 113 of whom had longitudinal outcome data. We examined associations between SCZ-PRS and diagnosis, clinical stage and functioning at initial assessment, and new-onset psychotic disorder, clinical stage transition and functional course over time in contact with services.

**Results:**

Compared with a control group, patients had elevated PRSs for schizophrenia, bipolar disorder and depression, but not for any non-psychiatric phenotype (for example cardiovascular disease). Higher SCZ-PRSs were elevated in participants with psychotic, bipolar, depressive, anxiety and other disorders. At initial assessment, overall SCZ-PRSs were associated with psychotic disorder (odds ratio (OR) per s.d. increase in SCZ-PRS was 1.68, 95% CI 1.08–2.59, *P* = 0.020), but not assignment as clinical stage 2+ (i.e. discrete, persistent or recurrent disorder) (OR = 0.90, 95% CI 0.64–1.26, *P* = 0.53) or functioning (*R* = 0.03, *P* = 0.76). Longitudinally, overall SCZ-PRSs were not significantly associated with new-onset psychotic disorder (OR = 0.84, 95% CI 0.34–2.03, *P* = 0.69), clinical stage transition (OR = 1.02, 95% CI 0.70–1.48, *P* = 0.92) or persistent functional impairment (OR = 0.84, 95% CI 0.52–1.38, *P* = 0.50).

**Conclusions:**

In this preliminary study, SCZ-PRSs were associated with psychotic disorder at initial assessment in a young, transdiagnostic, clinical cohort accessing early-intervention services. Larger clinical studies are needed to further evaluate the clinical utility of SCZ-PRSs, especially among individuals with high SCZ-PRS burden.

## Major developments in psychiatric genetics

The past decade has witnessed two major conceptual shifts in our understanding of the genetic architecture of mental disorders. First, in contrast to disorders caused by a single genetic variant (such as *huntingtin* in Huntington's disease^1^), genetic risk for mental disorders involves the contribution of hundreds or thousands of common variants of small effect^2^ and/or rare variants of larger effect (for example duplications, deletions).^3^ That is, they are ‘polygenic’.^4^ Notably, even classic monogenic disorders such as Huntington's disease^5^ and *BRCA1* breast cancer^6^ are recognised to have a polygenic component influencing age at onset.

Strong evidence of polygenicity in mental disorders has come from international collaborative psychiatric genome-wide association studies (GWASs).^2,7–10^ For example, major efforts by the Psychiatric Genomics Consortium (PGC) have identified 108 genetic loci associated with schizophrenia,^2^ 102 loci associated with major depression^11^ and 30 loci associated with bipolar disorder.^8^ A key implication of these studies is that the causal impact of single variants is likely to be small, and vulnerability for complex psychiatric phenotypes is associated with a high load of risk variants.

The second major conceptual shift is that the genetic architecture of the major mental disorder diagnoses overlaps across disorders. For example, the Cross-Disorder group of the PGC has provided direct molecular evidence of shared genetic risk, reporting high genetic correlations (based on single-nucleotide polymorphisms (SNPs)) between schizophrenia and bipolar disorder; and moderate genetic correlations between schizophrenia and depression; bipolar disorder and depression; and attention-deficit hyperactivity disorder (ADHD) and depression.^12^

A recent study of GWAS data on 25 common brain disorders supported the high degree of shared genetic risk among major mental disorders (ADHD, depression, bipolar disorder, schizophrenia), whereas neurological disorders such as generalised epilepsy, Alzheimer's disease, Parkinson's disease and multiple sclerosis were more genetically distinct, suggesting greater diagnostic specificity and/or distinct aetiologies for neurological disorders.^13^ These and other studies^14,15^ suggest that alterations in key biological pathways (for example neuronal, immune) are frequently shared across the major mental disorders, particularly during brain development.

## Polygenic risk scores in psychiatry

A recent tool emerging from psychiatric GWASs that captures features of these two conceptual shifts is the ‘polygenic risk score’ (PRS).^16^ A PRS is an estimate of an individual's genetic liability to a particular trait or phenotype, calculated as a weighted count of risk alleles, with the risk alleles and their weights derived from GWASs (noting that these individuals are unrelated to the samples included in the GWAS).^17^ Although the effect sizes of known variants are currently too small for outcome prediction using any single variant, the PRS framework allows incorporation of many variants of small effect (and is robust to inclusion of false positives) to produce an aggregate index of liability to disorder.

Although the variance in liability to schizophrenia explained by the PRS (SCZ-PRS) is only ~7%,^2^ several studies have examined the potential clinical utility of the SCZ-PRS. Several studies to date have shown that in samples with psychotic disorders, the SCZ-PRS is strongly and robustly associated with the diagnosis of schizophrenia,^18^ transition from clinical high risk or a different mental disorder (for example depression) to full-threshold psychotic disorder,^19–21^ poorer neurocognition^22,23^ and social cognition,^22^ negative symptoms^24^ and poorer illness course;^23,25^ however, it is important to note that some studies have not observed significant associations between the SCZ-PRS and aspects of illness course (such as treatment resistance),^26,27^ and impairments in overall neurocognition^28^ and specific neurocognitive domains.^29,30^ Finally, the SCZ-PRS has been observed to be positively associated with other mental disorders including depression, bipolar disorders, substance use disorders and anxiety disorders, among others (i.e. ‘genetic pleiotropy’).^18,31^

## Current study

The shared genetic risk across major mental disorders and pleiotropy of the SCZ-PRS begs the question of whether it could have utility for predicting outcomes in broader transdiagnostic samples.^32^ There has been a shift toward a recognition of transdiagnostic models of mental disorders that acknowledge the dynamic nature of syndrome-based phenotypes^33^ and their limited specificity to aetiology, genetic architecture, risk factors and neurobiology.^13,34–39^ Efforts to improve prediction of illness trajectories and outcomes is particularly important in young people in the early phases of mental disorders, during which syndromes and diagnoses are more plastic.^33,40–42^ Accordingly, this study aimed to evaluate the utility of PRSs in a transdiagnostic clinical cohort of adolescents and young adults accessing early-intervention mental health services. While PRSs were calculated for a range of psychiatric (for example depression, bipolar disorder) and non-psychiatric phenotypes (for example body mass index, peptic ulcer disease, type 2 diabetes, cardiovascular disease), the current study focuses on the schizophrenia PRS. Specifically, we aimed to examine associations between SCZ-PRS and diagnosis, clinical stage and functioning around the time of entry to clinical services, and between SCZ-PRS and new-onset psychotic disorder, clinical stage transition and persistent functional impairment over time in contact with clinical services.

## Method

### Human ethics and study reporting

The authors assert that all procedures contributing to this work comply with the ethical standards of the relevant national and institutional committees on human experimentation and with the Helsinki Declaration of 1975, as revised in 2008. All procedures involving human patients were approved by the University of Sydney Human Research Ethics Committee (2012/1626, 2012/1631). Written informed consent was obtained from all patients and/or their guardians. This study followed the Strengthening the Reporting of Observational Studies in Epidemiology (STROBE) reporting guideline.^43^

### Participants

Study participants were drawn from a large research case register of consecutive referrals to youth mental health clinics at the Brain and Mind Centre in Sydney, Australia between 2004 and 2018, and were recruited to a neurobiological study of the early phases of mental disorders.^44^ These clinics (such as ‘*headspace*’) provide highly accessible and youth-friendly early-intervention services for young people experiencing problems with substance use and/or mental health, attracting young people with a range of subthreshold and full-threshold mental health syndromes (commonly mood, anxiety and psychotic syndromes).^44^ *headspace* consists of an integrated mix of primary-level and specialist services, and participants were receiving clinician-based case management and relevant evidence-based social, psychological and/or medical interventions as part of standard clinical care, which may have involved contact with a psychiatrist, psychologist, occupational therapist, social worker or hospital admission.^44^

### Eligibility criteria

Eligibility criteria for this study were:

- an available SCZ-PRS;
- aged 12–30 at baseline;
- European ancestry; and
- willing/able to give informed consent (and/or parental consent was obtained).

Potential participants were excluded from the broader neurobiological study (and by extension this study) if they had:

- history of neurological disease;
- medical illness known to affect brain function (such as epilepsy);
- received electroconvulsive therapy in the 3 months prior to assessment;
- clinically determined intellectual disability (i.e. IQ < 70); and/or
- insufficient understanding of the English language to allow participation in verbal assessments/testing.

### Clinical and functional outcomes

The methodology used here is described in greater detail elsewhere.^44–47^ Briefly, trained research staff used a standardised clinical proforma to gather demographic, clinical and functioning data from research and clinical case files across eight predetermined time points. The proforma collects standardised information regarding:

- basic demographics (such as gender, age);
- subthreshold and full-threshold mental health diagnoses;
- clinical course (such as clinical stage, admission to hospital);
- comorbidities (such as physical health conditions); and
- functioning.

Phase I and II of data extraction of the ‘Optymise’ cohort concluded in 2019, and the cohort comprises 2901 participants from our clinical case register.^44^ In the current study, we focused on the following outcomes: mental disorder diagnoses, clinical stage and functioning.

#### Mental disorder diagnoses

Mental disorder diagnoses were classified according to DSM-5 criteria^48^ and labelled as either primary, secondary or tertiary based on judgement of which was the dominant presenting problem at the particular time point. Diagnosis was determined solely by diagnosis reported and recorded by the treating clinician(s) as presented in clinical notes or symptomatology. Based on information recorded in the clinical notes, researchers determined whether DSM-5 criteria were met for a disorder at that time point. If symptomatology recorded in the notes indicated some, but not all criteria being met for a disorder, then a subthreshold classification was recorded.

#### Clinical stage

Clinical stage was assigned according to an established model.^33,49,50^ Descriptions of the criteria for the stages within this model are detailed elsewhere,^49^ and a decision tree is available in ^44^. Briefly, individuals are assigned to one of six stages including: stage 0 (no current symptoms; increased risk of disorder); stage 1a (mild or non-specific symptoms); stage 1b (moderate but subthreshold symptoms); stage 2 (full-threshold disorder with moderate to severe symptoms); stage 3 (incomplete remission or relapse); or stage 4 (severe, unremitting or refractory illness).^33^

#### Functioning

Functioning was measured by the clinician-rated Social and Occupational Functioning Assessment Scale (SOFAS).^51^ The SOFAS is a 100-point scale (higher scores denoting better functioning), with instructions to raters to avoid confounding the rating of functioning with symptoms. A SOFAS score of below 70 is considered to indicate clinically significant impairment.^52^

### PRSs

A subset (*n* = 193) of the cohort had blood collected and genotyped at the Queensland Institute for Medical Research Berghofer Molecular Epidemiology Laboratory using the Illumina Psych Chip v1.0 under standard protocols. Stringent quality control procedures were implemented in GenomeStudio and Plink2 and applied to these data plus an independently collected control sample. This independent control sample (*n* = 1528) comprised unaffected Australians who were genotyped in case–control studies of motor neuron disease and Parkinson's disease (details available from the authors on request).^53^ Briefly, SNPs were filtered for call missingness >10%, departure from Hardy–Weinberg equilibrium (*P* < 10^−6^), minor allele frequency < 0.01, and deviation from allele frequency compared with the Haplotype Reference Consortium.^54^ After quality control, genotyped SNPs were submitted to the Sanger Imputation Server for imputation to the Haplotype Reference Consortium reference samples. Using the PC projection method, the samples were projected to the 1000Genome reference samples,^55^ and then assigned to a population if they clustered with the population within 3 s.d.s.

Among the 193 clinical samples, 161, 10 and 22 were assigned European, East Asian and other ancestry, respectively, based on genetic data. Here, we focus on participants of European ancestry. PRSs were generated for eight traits using SBayesR:^56^ three mental disorders/traits (schizophrenia,^57^ bipolar disorder,^8^ depression^11^), height,^58^ and four traits of relevance to common comorbidities of mental disorders (body mass index,^58^ cardiovascular disease,^59^ type 2 diabetes,^59^ peptic ulcer disease^60^). The PRSs of the control and clinical participants were standardized by subtracting the mean and dividing by the s.d. of the control sample. Here, we focus on the SCZ-PRS based on it having the largest GWAS discovery sample in psychiatry,^2^ and to limit the number of statistical tests performed.

### SCZ-PRS

The PRS comparing the European ancestry clinical sample with the independent control sample (*n* = 1528) has been reported previously.^61^ The PRSs are scaled to have a mean of 0 and a s.d. of 1 in a population sample. Briefly, and to illustrate the elevated psychiatric PRSs in the young clinical sample, the difference in mean SCZ-PRS between the clinical sample and controls was 0.54 control s.d. units (*P* = 1.6 × 10^−10^), 0.29 control s.d. units for bipolar disorder (*P* = 5.1 × 10^−4^), and 0.46 control s.d. units for depression (*P* = 6.2 × 10^−8^). In contrast for the non-psychiatric traits of height, body mass index, coronary artery disease, type 2 diabetes and peptic ulcer disease, the differences between mean PRS for clinical participants and controls were non-significant.

In the following analyses, we focus only on the SCZ-PRS in those of European ancestry for three reasons. First, the GWAS used to generate the PRS is the largest. In terms of variance explained on the liability scale, the SCZ-PRS explains at least 7%, whereas the respective PRSs for bipolar disorder and depression explain 4% of each disorder.^8,62^ Second, mental disorders are genetically correlated. For example, the genetic correlation between schizophrenia and bipolar disorder is high (~0.65),^12^ and hence PRSs for these disorders will be correlated. Third, a high SCZ-PRS likely represents a genetic risk for mental disorders that is not specific for schizophrenia. Importantly, a high SCZ-PRS in the context of help-seeking young people could be useful in clinical decision-making.^61^

### Statistical analysis

Analyses were performed using R statistical software with the RStudio IDE.^63^ Continuous data are summarised as means and s.d.s, and categorical data are summarised as frequencies and percentages. Linear regression was used for the continuous outcome and logistic regression for binary outcomes. As the SCZ-PRS is in s.d. units of the control group, odds ratios (OR) are interpretable as a 1 s.d. increase or decrease in the SCZ-PRS. Data were missing for < 5% of participants for each variable (Supplementary Table 1 available at https://doi.org/10.1192/bjo.2021.14) and all analyses were on ‘complete cases’.

## Results

### Participant characteristics

Of the 2901 participants in the Optymise cohort, 193 patients were genotyped, and a total of 158 patients met all eligibility criteria. Characteristics of the final participants are in Table 1.

**Table 1:** Baseline characteristics of 158 young people of European ancestry presenting to mental health clinics

	Values
Age, years: mean (s.d.)	20.7 (4.7)
Gender, female: *n* (%)	59 (37.3)
Social and Occupational Functioning Assessment Scale, mean (s.d.)	58.4 (10.2)
Clinical stage,^a^ *n* (%)	
1a – ‘non-specific symptoms’	22 (13.9)
1b – ‘attenuated syndrome’	85 (53.8)
2 – ‘discrete disorder’	33 (20.9)
3 – ‘recurrent or persistent disorder’	14 (8.9)
4 – ‘persistent, chronic and unremitting’	2 (1.3)
Primary diagnosis,^b^ *n* (%)	
Anxiety disorder	15 (9.5)
Bipolar disorder	22 (13.9)
Depressive disorder	60 (38.0)
Psychotic disorder	26 (16.5)
Other	34 (21.5)

a. Missing for *n* = 2.

b. Missing for *n* = 1.

At baseline, the participants comprised 158 young people accessing youth mental health services; 99 were male (62.7%) and 59 were female (37.3%), with a mean age of 20.7 (s.d. = 4.7) years at baseline (range 12–30). Around half the participants presented as stage 1b (53.8%, *n* = 85) and around one-fifth presented as stage 2 (20.9%, *n* = 33) (see Table 1 for clinical stage ratings). The majority of the participants presented with a primary mood (depressive or bipolar disorder) or anxiety syndrome (61.4%, *n* = 97) and around one-sixth presented with a primary psychotic syndrome (16.5%, *n* = 26).

Functional impairment was common, with a mean clinician rating of functioning on the SOFAS of 58.4 (s.d. = 10.2), falling in the band ‘moderate difficulty in social, occupational or school functioning’. A total of 113 participants had longitudinal data (71.5%), with a mean follow-up duration of 40.8 (s.d. = 30.5) months.

Compared with participants with only baseline data, those with follow-up data were on average younger (20.0 *v.* 22.5 years old; *P* < 0.002) and there was a trend toward more females (42.5% *v.* 24.4%; *P* = 0.053). There was no difference in functioning as measured by the SOFAS (58.0 *v.* 59.7; *P* = 0.354).

Numerical differences in proportions of diagnoses and clinical stages are shown in Supplementary Table 2.

### Associations between SCZ-PRS and diagnosis, clinical stage and functioning

#### SCZ-PRS and diagnosis

**Fig. 1:**
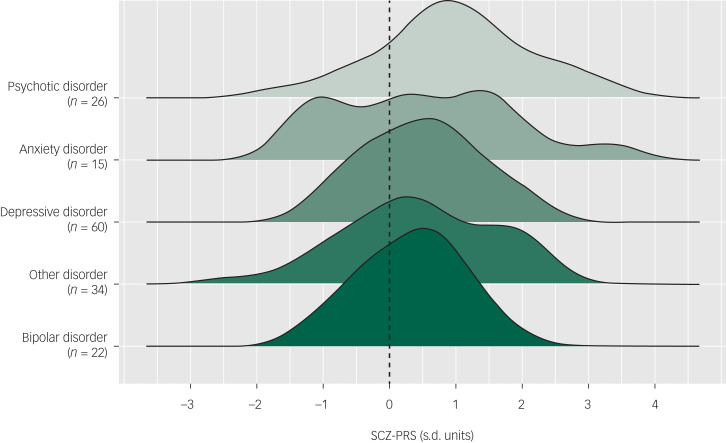
Distributions of schizophrenia polygenic risk scores (SCZ-PRSs) by primary diagnoses at baseline, ordered by mean SCZ-PRSs (dashed line represents mean of control sample).

At baseline, the pattern of SCZ-PRSs across primary diagnoses (i.e. the diagnosis identified as the main presenting problem) was highest among those with a psychotic disorder (mean 0.97, s.d. = 1.15, *n* = 26), followed by anxiety disorder (mean 0.51, s.d. = 1.35, *n* = 15), depressive disorder (mean 0.49, s.d. = 0.85, *n* = 60), other disorder (mean  0.45, s.d. = 1.12, *n* = 34) and bipolar disorder (mean 0.32, s.d. = 0.75, *n* = 22). The distributions of SCZ-PRS across disorders are shown in Fig. 1. Logistic regression showed that higher overall SCZ-PRS was associated with the presence of a primary psychotic disorder at baseline (OR = 1.68, 95% CI 1.08–2.59, *P* = 0.020).

Among the 113 participants with longitudinal data, five participants (~4% of the follow-up group) had a new-onset psychotic disorder (i.e. no psychotic disorder at baseline but incidence of psychotic disorder over follow-up). Participants who developed a new-onset psychotic disorder had numerically lower SCZ-PRSs (mean 0.28, s.d. = 2.01) than participants who had no psychotic disorder at baseline or over follow-up (mean 0.42, s.d. = 0.95). We did not observe a significant association between SCZ-PRS and new-onset psychotic disorder over follow-up (OR = 0.84, 95% CI 0.34–2.03, *P* = 0.69).

#### SCZ-PRS and clinical stage

**Fig. 2:**
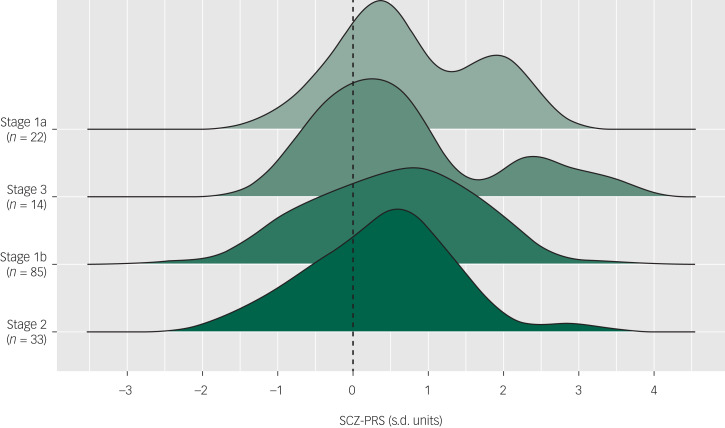
Distribution of schizophrenia polygenic risk scores (SCZ-PRSs) by clinical stage at baseline, ordered by mean SCZ-PRSs (dashed line represents mean of control sample). Stage 4 not displayed as *n* < 3.

At baseline, the pattern of SCZ-PRSs was highest among those assigned stage 1a (mean 0.75, s.d. = 0.91, *n* = 22), followed by stage 3 (mean 0.68, s.d. = 1.20, *n* = 14), stage 1b (mean 0.52, s.d. = 1.04, *n* = 85), stage 2 (mean 0.38, s.d. = 0.93, *n* = 33) and stage 4 (mean 0.34, s.d. = 0.34, *n* = 2). Of note, the SCZ-PRS distributions were highly right-skewed for participants assigned stage 3, suggesting an overrepresentation of individuals with high SCZ-PRS scores (Fig. 2). However, a logistic regression did not show an association between overall SCZ-PRS and assignment at stage 2+ at baseline (OR = 0.90, 95% CI 0.64–1.26, *P* = 0.53).

Among those with follow-up data (*n* = 113), a total of 47 participants transitioned from a lower to a higher clinical stage over time in care. SCZ-PRSs were numerically similar among individuals who transitioned from a lower to a higher clinical stage (mean 0.44; s.d. = 1.01) compared with those who did not transition (mean 0.46; s.d. = 1.03). Logistic regression showed that the SCZ-PRS was not significantly associated with transition from a lower to a higher clinical stage (OR = 1.02, 95% CI 0.70–1.48, *P* = 0.92) or from a subthreshold clinical stage (1a or 1b) to a full-threshold (2+) clinical stage (OR = 0.98, 95% CI 0.66–1.44, *P* = 0.90).

#### SCZ-PRS and social and occupational functioning

**Fig. 3:**
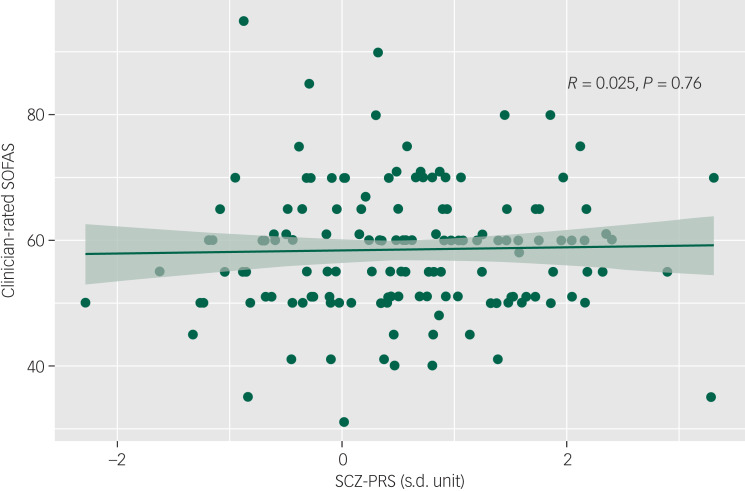
Association of schizophrenia polygenic risk score (SCZ-PRS) and baseline social and occupational functioning among young people accessing mental health services. SOFAS, Social and Occupational Functioning Assessment Scale.

A linear regression showed that SCZ-PRS was not significantly associated with baseline functioning (SOFAS) (*R* = 0.03, *P* = 0.76) (Fig. 3). Among those with at least one follow-up time point, SCZ-PRS was also not significantly associated with being functionally impaired (i.e. SOFAS <70) across two time points (OR = 0.84, 95% CI 0.52–1.38, *P* = 0.50).

### Exploratory analysis

In an exploratory analysis, we examined the characteristics of participants who had a SCZ-PRS of ≥1.64 (wherein ~10% of the control participants are expected to fall) compared with a SCZ-PRS below 1.64. We show in Table 2 that there were numerically higher proportions of participants in the ‘high’ SCZ-PRS group (≥1.64) compared with the ‘low’ SCZ-PRS group (<1.64) who had a psychotic disorder at baseline (22.7% *v.* 15.4%) or a new-onset psychotic disorder over follow-up (7.1% *v.* 4.0%).

**Table 2:** Key characteristics of individuals with a ‘high’ schizophrenia polygenic risk score (SCZ-PRS, ≥1.64) and ‘low’ SCZ-PRS (<1.64)

	‘High’ (≥1.64) SCZ-PRS (*n* = 22)	‘Low’ (<1.64) SCZ-PRS (*n* = 136)
Age, years:	20.1 (3.7)	20.8 (4.8)
Gender, female: *n* (%)	6 (27.3)	53 (39.0)
Baseline psychotic disorder, *n* (%)	5 (22.7)	21 (15.4)^a^
New-onset psychotic disorder,^b^ *n* (%)	1 (7.1)	4 (4.0)^c^
Clinical stage transition,^b^ *n* (%)	4 (28.6)	43 (43.4)^c^

a. Missing for *n* = 1.

b. Participants with follow-up data: *n* = 113.

c. Missing for *n* = 1.

## Discussion

### Principal findings

To our knowledge, this is the first study to examine the clinical utility of the SCZ-PRS in a transdiagnostic clinical cohort of adolescents and young adults in the early phases of mental disorders. We observed an association between higher overall SCZ-PRS and diagnosis of a psychotic disorder at baseline, supporting the link between the SCZ-PRS and liability towards psychotic disorders such as schizophrenia.^18^ We did not find evidence of a significant relationship between the SCZ-PRS and functioning or assignment at clinical stage 2+ at baseline. Although underpowered, our longitudinal analyses did not detect associations between the SCZ-PRS and an impaired course of functioning, nor for incidence of psychotic disorder or clinical stage transition over follow-up. However, these non-significant findings are not surprising given the patterns of associations at baseline. Of note, SCZ-PRS were higher in all patient diagnostic groups compared to the control participants, consistent with recent findings of pleiotropic effects of the SCZ-PRS on other non-psychotic mental disorders.^18^

### SCZ-PRSs and psychotic disorder

Numerous studies to date have demonstrated an association between the SCZ-PRS and schizophrenia,^2,64,65^ with some also reporting associations with a broader spectrum of disorders including schizoaffective disorder, psychotic disorder not otherwise specified and bipolar I disorder.^64,66^ Notably, the OR of SCZ-PRSs on presence of a psychotic disorder at baseline in the current study (OR = 1.68) is similar to that reported for SCZ-PRSs and schizophrenia in a recent large study across four US healthcare systems (OR = 1.55).^18^ A recent study examined whether the SCZ-PRS can be used to predict incident psychotic disorders, reporting modest improvement of prediction of conversion to schizophrenia with the addition of the SCZ-PRS to an existing risk calculator among individuals at clinical high risk of schizophrenia.^19^ Although we did not observe a significant association between the SCZ-PRS and incidence of a new-onset psychotic disorder over follow-up, larger studies will be needed to more conclusively determine whether this type of prediction has utility beyond high-risk cohorts.

### SCZ-PRS and social and occupational functioning

As the SCZ-PRS has been reported to be associated with severity of neurocognitive impairment,^22,23^ negative symptoms^24^ and poorer course of illness defined by the Global Assessment of Functioning,^23^ we speculated that the SCZ-PRS might be related to functional impairment. However, our findings did not offer support for such a relationship.

### SCZ-PRS and clinical stage

Several studies have suggested that the SCZ-PRS is associated with a poorer course of illness, namely chronic^25^ and treatment-resistant schizophrenia.^67-69^ Accordingly, we wondered whether the SCZ-PRS might also be associated with a more severe illness course transdiagnostically, as determined by greater stage of illness in a transdiagnostic clinical staging model.^32,33,70^ However, our analyses did not show an association for assignment at clinical stage 2+ at baseline. Similarly, the SCZ-PRS was not significantly associated with transition to a more advanced clinical stage over follow-up.

Two qualifying points are worth noting. First, of the 49 participants who were assigned stage 2+ at baseline, only around one-third had a psychotic disorder (*n* = 19, 38.8%). Second, of the 38 participants that transitioned to a stage 2+ disorder over follow-up, almost three-quarters transitioned to non-psychotic disorders (71.1%, *n* = 27). Two potential interpretations of these findings are that the SCZ-PRS may not be associated with transition to more advanced stages of non-psychotic disorders, or alternatively, the SCZ-PRS may not be robustly associated with course of psychotic disorders more specifically, as suggested by some studies.^26,27^ Larger clinical studies with greater statistical power will be needed to clarify these points.

### Limitations

Several limitations should be mentioned. First, the SCZ-PRS were derived from a European ancestry discovery sample, and these scores have been reported to have poorer accuracy in non-European ancestries.^71^ Although we focused our main analyses on participants with European ancestry, it is worth noting that the East Asian ancestry group had substantially higher SCZ-PRS scores (Supplementary Fig. 1). Speculatively, there may be important cultural factors influencing thresholds for help-seeking in this East Asian ancestry group. For example, some individuals may ‘require’ very severe illness (and possibly high polygenic burden) in order to cross thresholds for seeking care. Importantly, all of our findings were robust to sensitivity analyses including European, East Asian and other ancestries. Nonetheless, ancestrally diverse GWASs are critical to achieve more generalisable and equitable PRSs.^71^

Second, the SCZ-PRS reflects variation captured by individual SNPs of small effect and does not capture rare SNPs or *de novo* mutations of larger effect (such as copy number variants, deletions). Third, and critically, the SCZ-PRS used in this study captures ~7% of the genetic liability to schizophrenia, and as such, larger GWAS studies are needed to increase the predictive power of the SCZ-PRS in clinical contexts.

Fourth, for reasons related to sample size, we focused our analyses on the overall SCZ-PRS (analysed as a continuous variable). A recent editorial^61^ has, however, suggested that an optimal use of the SCZ-PRS may be to focus prediction efforts on a select subgroup with high SCZ-PRSs (as in our exploratory analysis; see Table 2), for whom this information may influence clinical decision-making. Larger clinical studies with higher statistical power are needed to better understand these relationships.

Fifth, the subset of the cohort who were genotyped were not randomly selected, and our results may not be fully generalisable to the broader help-seeking populations accessing transdiagnostic youth mental health services. Relatedly, data regarding the quantity, quality, and intensity of treatment and engagement was not systematically captured, and it is possible that heterogeneity in treatment patterns may confound some of our findings. Finally, incidence of psychotic disorder over follow-up was a relatively rare event (*n* = 5; ~4% longitudinal sample) and larger studies are needed to better answer the question of whether the SCZ-PRS can predict new cases and tilt clinical decision-making.^72^

### Future directions

The SCZ-PRS will very likely be improved in coming years with the addition of novel SNPs identified in larger GWASs and may be further strengthened by inclusion of rarer genetic variants with larger effects. Critically, these developments may improve the predictive power of the SCZ-PRS and support its inclusion in clinical decision-making. Larger clinical studies focusing on subgroups with high SCZ-PRSs will be crucial for testing this hypothesis.

## Data Availability

The data that support the findings of this study are available on reasonable request from the corresponding author, J.J.C. The data are not publicly available due to their containing information that could compromise the privacy of research participants.

## Supplementary material

For supplementary material accompanying this paper visit http://dx.doi.org/10.1192/bjo.2021.14. click here to view supplementary material
