# Supplementary material for: Schizophrenia polygenic risk scores in youth mental health: preliminary associations with diagnosis, clinical stage and functioning
Source: BJPsych Open. 2021 Feb 22;7(2):e58. doi: 10.1192/bjo.2021.14 (PMC8058892; doi:10.1192/bjo.2021.14)
Supplement: Supplementary file 1 [file bjosup.zip › S2056472421000144sup001.docx]

**Supplementary Table 1.** Missing data for key variables for final sample (N=158).

|  | **Baseline (N=158)** | **Longitudinal (N=113)** |
| --- | --- | --- |
|  | **N (%)** | **N (%)** |
| Age, years | 0 (0.0) | 0 (0.0) |
| Sex | 0 (0.0) | 0 (0.0) |
| Primary diagnosis | 1 (0.6) | 1 (0.9) |
| Clinical stage | 2 (1.3) | 1 (0.9) |
| SOFAS | 7 (4.4) | 4 (3.5) |

***Note***: SOFAS = Social and Occupational Functioning Assessment Scale

**Supplementary Table 2.** Characteristics of participants with available longitudinal data and those without.

|  | **Baseline only (N=45)** | **Longitudinal**  **(N=113)** | **Statistical test** | |
| --- | --- | --- | --- | --- |
|  | **M (SD) or N (%)** | **M (SD) or N (%)** | **t** | **P** |
| Age, years | 22.5 (4.5) | 20.0 (4.6) | 3.21 | 0.002 |
| Sex (female) | 11 (24.4) | 48 (42.5) | 3.74 | 0.053 |
| Primary diagnosis ^a^ |  |  |  |  |
| Depressive disorder | 18 (40.0) | 42 (37.2) | — | — |
| Anxiety disorder | 2 (4.4) | 13 (11.5) | — | — |
| Bipolar disorder | 6 (13.3) | 16 (14.2) | — | — |
| Psychotic disorder | 11 (24.4) | 15 (13.3) | — | — |
| Other | 8 (17.8) | 26 (23.0) | — | — |
| Clinical stage ^a^ |  |  |  |  |
| 1a | 5 (11.1) | 17 (15.0) | — | — |
| 1b | 21 (46.7) | 64 (56.6) | — | — |
| 2 | 9 (20.0) | 24 (21.2) | — | — |
| 3 | 7 (15.6) | 7 (6.2) | — | — |
| 4 | 1 (2.0) | 1 (0.9) | — | — |
| SOFAS | 59.7 (11.7) | 58.0 (9.6) | 0.93 | 0.354 |

Note: ^a^ = Chi-square tests were not conducted for these variables; SOFAS = Social and Occupational Functioning Assessment Scale
