# Supplementary material for: Schizophrenia polygenic risk scores in youth mental health: preliminary associations with diagnosis, clinical stage and functioning
Source: BJPsych Open. 2021 Feb 22;7(2):e58. doi: 10.1192/bjo.2021.14 (PMC8058892; doi:10.1192/bjo.2021.14)
Supplement: Supplementary file 1 [file bjosup.zip › S2056472421000144sup003.docx]

**Supplementary Figure 1. Distribution of SCZ-PRS scores across European (EUR), East Asian (EAS), and other ancestries.**

**
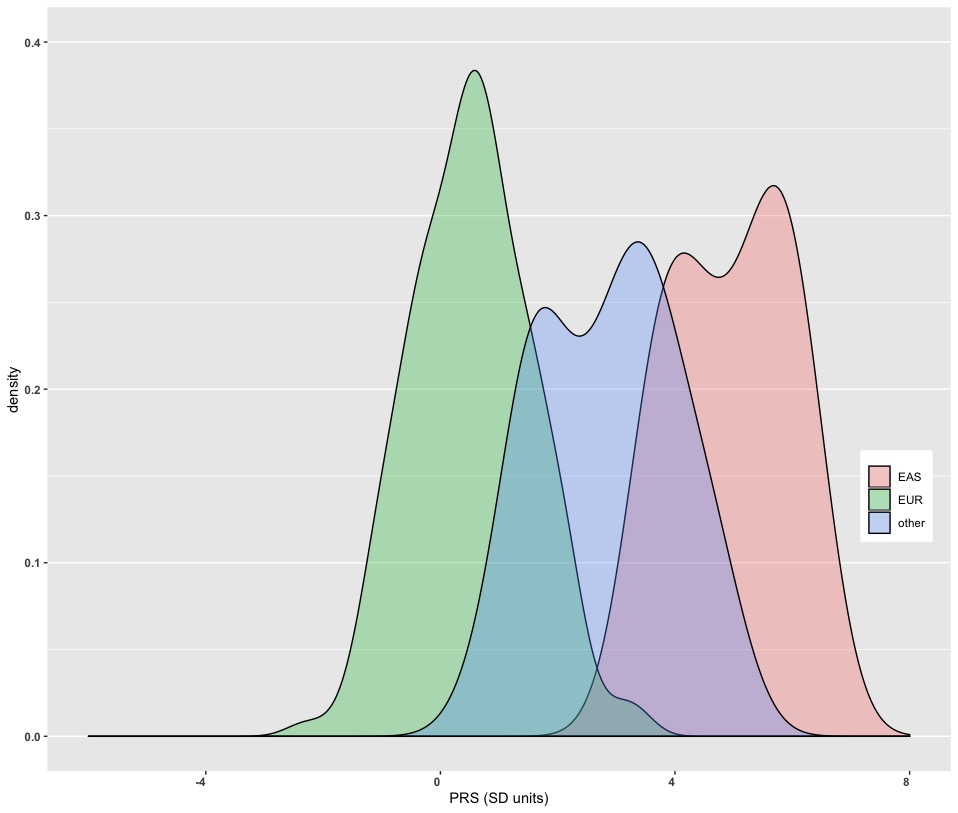
**
